# An observational study to compare the treatment outcomes of older and newer antiretroviral regimen in HIV patients

**DOI:** 10.6026/973206300220098

**Published:** 2026-01-31

**Authors:** Lovely Lahare, Shashi Marko, Lily Dubey, Pawan Gupta

**Affiliations:** 1 Department of Pharmacology, Bundelkhand Medical College, Sagar, Madhya Pradesh, India; 2 Department of Dermatology, Bundelkhand Medical College, Sagar, Madhya Pradesh, India

**Keywords:** Dolutegravir, CD4 count, viral load, antiretroviral therapy

## Abstract

The need to compare the effectiveness and adverse effects of Dolutegravir-based regimens versus older TLE regimens in HIV treatment is relevant. Therefore, it is of interest to evaluate their comparative outcomes in a clinical setting including 180 adults with HIV. Results showed comparable efficacy in CD4 counts and viral load between the two groups, although Dolutegravir seemed to cause a quicker increase in CD4 counts. This study contributes to the existing body of knowledge by providing direct comparisons of Dolutegravir-based and older TLE regimens in HIV treatment, highlighting both efficacy and safety outcomes, with a specific focus on CD4 count progression and adverse effects.

## Background:

HIV is a global health problem with the prevalence of 39.9 million Patients Living with HIV infection as per the World Health Organization (WHO) report published in 2023 [1]. In India, the prevalence is 0.2%, equating to 2.54 million PLHIV, as per the 2023 projections from the National AIDS Control Organization (NACO) [2]. Advancements in Antiretroviral therapy (ART) have transformed HIV management, enabling PLHIV to achieve near-normal life expectancy with sustained viral suppression and immunological recovery [3]. The evolutions of ART regimens have been essential to optimise efficacy, safety and resistance profiles in accordance with the disease population [4]. As per the National HIV Care and Management Recommendations 2021, a Dolutegravir-based regimen is recommended as the initial antiviral therapy for individuals with PLHIV compared to the earlier tenofovir + lamivudine + Efavirenz (TLE) regimen [5]. The Dolutegravir-based regimen comprises of Tenofovir, Lamivudine and Dolutegravir (TLD). Dolutegravir is an Integrase Strand Transfer Inhibitor (INSTI) which provides several pharmacological advantages over the conventional regimen which includes quick viral suppression, lower failure rates, minimal drug-drug interactions and better efficacy in HIV-2 and co-infections such as tuberculosis (TB) and hepatitis B virus (HBV). The previous regimen included Efavirenz which is prone to cause hepatotoxicity and neuropsychiatric symptoms [6,7]. Therefore, it is of interest to determine the comparative effectiveness and safety of Dolutegravir-based regimens, providing valuable insights for optimizing HIV treatment strategies.

## Materials and Methods:

This observational Cross-sectional study was conducted in ART centre, Bundelkhand Medical College, Sagar, Madhya Pradesh, after obtaining institutional ethics committee approval (IECBMC/DHR/2024/52 Dated 26.02.24). Details about the research participants diagnostic and demographics attributes were extracted from the ART register and ART card, collected on a predesigned case record form and transformed into a Microsoft excel sheet, analysed using SPSS v26. The inclusion criteria for the study participants was ≥ 18 years of age, treatment naive adult patients diagnosed with HIV infection and initiated on first-line ART. Patient records lacking essential data in the ART register or card were excluded from the analysis, including those with missing files, incomplete details, unrecorded outcomes, or cases transferred out. The study duration was 1 year. The efficacy parameters evaluated in the study were viral load suppression and CD4 counts. These parameters were assessed at baseline visit and after 6 and 12 months respectively, following the initiation of antiretroviral treatment (ART). CD4 count was categorized as <200 cells/mm^3^ or >200 cells/mm^3^. The study included covariates related to socio-demographic and clinical factors. Socio-demographic factors encompassed age, gender, place of residence, marital status, education level and mode of transmission. Clinical factors encompassed laboratory parameters and drug-related factors included adherence to the treatment, drug substitution, occurrence of adverse effects and opportunistic-infections. A paired student's t-test was employed to compare CD4 cell counts, weight and virologic levels at follow against their baseline values. An independent t-test was used to evaluate differences in mean weight, CD4 count increase and viral suppression levels at six months between two ART regimens. P-value criteria of less than 0.05 and a 95% confident range were used to establish statistically relevance.

## Results:

The study included 180 patients, with 93 (51.7%) females, and the most common mode of HIV transmission was sexual (177, 98.3%). The patients were divided into two groups: Regimen 1 (TLD) and Regimen 2 (TLE). The mean age of patients in Regimen 1 was 28.63 ± 5.09 years, while in Regimen 2 it was 31.40 ± 7.89 years, with no significant difference between the groups (p = 0.160). The gender distribution was similar across both groups, with 51.1% females in Regimen 1 and 52.2% females in Regimen 2 (p = 0.881). At baseline, the mean weight for Regimen 1 was 51.44 ± 10.96 kg, and for Regimen 2, it was 52.93 ± 11.66 kg, with no significant difference (p = 0.379). Weight changes across visits were also similar between the groups (p values for visit 1 and visit 2 were 0.550 and 0.597, respectively). Haemoglobin levels at baseline were 11.9 ± 2.1 gm/dL for Regimen 1 and 11.5 ± 2.3 gm/dL for Regimen 2 (p = 0.218), and no significant differences were observed across subsequent visits. For serum creatinine, the baseline levels were slightly higher in Regimen 1, but the differences were not statistically significant (p = 0.308). At visit 1, 7.8% of Regimen 1 patients and 12.2% of Regimen 2 patients had elevated serum creatinine (p = 0.457), and at visit 2, 4.4% and 6.7%, respectively (p = 0.747). SGOT and SGPT levels exceeded the 3ULN threshold in both regimens at baseline and subsequent visits, but the differences were not statistically significant. The baseline CD4 count was significantly lower in Regimen 1 (27.8% with CD4 <200 cells/mm^3^) compared to Regimen 2 (10%, p = 0.0023) and by visit 2, Regimen 1 showed no patients with CD4 <200 cells/mm^3^, while Regimen 2 had 8.33% (p = 0.0031). Regarding viral load, there were no significant differences between the groups at baseline or at visits 1 and 2, with p-values ranging from 0.196 to 0.989. These results suggest that while there are some differences in CD4 count and other biomarkers, most of the demographic and disease characteristics between the two regimens were similar (Table 1). The details of demographic and disease characteristics in the two groups is shown in detail in Table 1. Serum urea was noticeably higher in older regimen in visit 1 p=0.047. Serum creatinine was noticeably higher in older regimen in visit 1 p=0.04. As per the latest guidelines, it is advisable to collect information regarding drug adherence from each patient administered HIV therapy. Hence, we collected data to calculate drug adherence. Also the change of any drug in the regimen, called as drug substitution was also noted. Detailed results pertaining to patient adherence, occurrence of adverse effects, and drug substitutions performed are recorded in Table 2.

## Discussion:

In current study, at baseline, 27.8% of TLD patients had CD4 <200 cells/mm^3^, compared to 10% of TLE patients. By the second visit, no TLD patients (0%) had CD4 <200 cells/mm^3^. In comparison, 8.33% of TLE patients did, similarly enhanced CD4 recovery in TLD cohort, increases from 6 to 36 months, and a higher proportion achieving CD4>200 by week 48th (29% TLE, 45%TLD) compared to TLE. Viral load (>1000 copies/mL) showed no significant differences at any time point in current study whereas, Viral suppression rates were consistently higher with TLD, ranging from 65.2% to 94.7% at follow-up periods of 12 to 48 weeks (4, 5 and 6), compared to 45.7% to 91.8% for TLE. The current study reported adverse effects in 8 TLD patients (5 experiencing weight gain) and 5 TLE patients. Due to impaired renal function (Tenofovir), drug substitution was needed for 3 TLD patients and 2 TLE patients. Opportunistic infections occurred in 15 TLD patients and 18 TLE patients. Additionally, TLD was associated with fewer adverse events, including lower discontinuation rates (4.2%TLD, 28.9%TLE), reduced treatment modifications (1%TLD, 17%TLE) and slightly lower dyslipidemia incidence (36%TLE, 31.3%TLD). The findings from various studies highlight key differences and similarities in the effectiveness and adverse events associated with TLE and TLD regimens Echefu *et al.* (2023) [8] found lower viral loads in TLD but no significant differences in hematological parameters. Zhong *et al.* (2023) [9] reported higher viral suppression at month 1 for TLD, with TLD showing a greater CD4 increase from months 6 to 36 and fewer treatment discontinuations compared to TLE. Li *et al.* (2023) [10] found improved viral suppression in both regimens, with mild adverse events in TLD.

## Conclusion:

DTG based regimen appear to be equally/better efficacious as the previous regimen presenting fewer adverse effects. Further studies with a greater number of participants and extended timeframe are necessary to validate these observations.

## Figures and Tables

**Table 1:** Comparison of demographic and disease characteristics in the two groups

**Study parameter (mean± SD)**	**Regimen 1 (TLD)**	**Regimen 2 (TLE)**	**P value**
Age (years)	28.63± 5.09	31.40 ± 7.89	0.16
Gender, females (N, %)	46 (51.1%)	47 (52.2%)	0.881
Weight baseline (kg)	51.44± 10.96	52.93± 11.66	0.379
Weight visit 1 (kg)	55.53± 12.31	54.44± 11.91	0.55
Weight visit 2 (kg)	57.53± 13.33	56.49± 12.49	0.597
Haemoglobin visit baseline (gm/dl)	11.9 ± 2.1	11.5± 2.3	0.218
Haemoglobin visit 1(gm/dl)	12.8± 2.1	12.2± 2.3	0.138
Haemoglobin visit 2 (gm/dl)	13.1± 2.1	12.6± 2.4	0.213
Serum creatinine baseline (mg/dL)	11 (12.2%)	6 (6.7%)	0.308
Serum creatinine visit 1 (mg/dL) *	7 (7.8%)	11 (12.2%)	0.457
Serum creatinine 2 (mg/dL)	4 (4.4%)	6 (6.7%)	0.747
*SGOT baseline (U/L) >3ULN	4 (4.4%)	3 (3.3)	0.5
SGOT visit 1(U/L) >3ULN	4 (4.4%)	3 (3.3)	0.5
SGOT visit 2 (U/L) >3ULN	3 (3.3)	1 (1.1%)	0.62
SGPT baseline (U/L) >3ULN	6 (6.7%)	5 (5.6%)	0.756
SGPT visit1 (U/L) >3ULN	4 (4.4%)	9 (10%)	0.249
SGPT visit 2 (U/L) >3ULN	3 (3.3)	2 (2.2%)	1
CD4 count baseline (<200cells/mm^3^) %	27.80%	10%	0.0023
CD4 count visit1 (<200cells/mm^3^) %	3.33%	8.33%	0.2426
CD4 count visit 2 (<200cells/mm^3^) %	0%	8.33%	0.0031
Viral load baseline (>1000 copies/ml) %	27.70%	22.22%	0.989
Viral load visit 1 (>1000 copies/ml) %	11.25%	6.89%	0.284
Viral load visit 2(>1000 copies/ml) %	10%	16.25%	0.196

**Table 2:** Details of patient adherence, adverse effects and drug substitution

**Study parameter**	**Regimen 1 (TLD)**	**Regimen 2 (TLE)**	**Remark**
Adherence percentage (>95%) visit 1	71 (78.8%)	56 (62.22%)	0.014
Adherence percentage (>95%) visit 2	56 (62.22%)	54 (60%)	0.75
Drug substitution (Tenofovir)	3	2	impaired RFT
Opportunistic infections	15	18	
Adverse effects	8 (weight gain -5)	5	
